# Cardiovascular Comorbidities in Knee Osteoarthritis: Prevalence, Functional association, and Therapeutic Consequences

**DOI:** 10.7759/cureus.116014

**Published:** 2026-09-09

**Authors:** Zeineb Alaoui Kadiri, Imane El Binoune, Bouchra Amine, Anbar Kabbaj, Ghizlane Ghoullam, Salma Zemrani, Samira Rostom, Rachid Bahiri

**Affiliations:** 1 Rheumatology A Department, El Ayachi Hospital, Mohammed V University, Salé, MAR

**Keywords:** cardiovascular comorbidities, knee osteoarthritis/ koa, knee pain, lequesne index, nsaid

## Abstract

Background: Knee osteoarthritis (KOA) is a common chronic degenerative joint disease and a leading cause of pain, disability, and impaired quality of life. Cardiovascular comorbidities (CVCs), particularly hypertension, dyslipidemia, and diabetes, frequently coexist with KOA and may influence both disease burden and treatment options, especially the use of non-steroidal anti-inflammatory drugs (NSAIDs). This study aimed to determine the prevalence of CVCs in patients with KOA and evaluate their association with pain, functional impairment, and therapeutic management.

Methods: We conducted a cross-sectional study including consecutive patients with radiographically confirmed KOA attending the Rheumatology A outpatient clinic at El Ayachi Hospital, Rabat-Salé, Morocco. Adults with inflammatory arthritis, previous major knee surgery, or post-traumatic osteoarthritis were excluded. Data collected included age, sex, disease duration, pain assessed by the visual analog scale (VAS), functional disability measured by the Lequesne index, cardiovascular comorbidities (hypertension, dyslipidemia, diabetes, coronary or vascular disease), and current treatments. Patients were divided into two groups according to the presence or absence of at least one cardiovascular comorbidity. Comparisons were performed using the chi-square test or Student's t-test, with p < 0.05 considered statistically significant.

Results: A total of 158 patients were included (mean age 62.3 ± 10.5 years; 125 (79.1%) women), with a median disease duration of seven years (IQR 4-12). Overall, 98 (62.0%) had at least one cardiovascular comorbidity. Hypertension was the most frequent (n=85; 53.8%), followed by diabetes mellitus (n=46; 29.1%) and dyslipidemia (n=41; 25.9%). Patients with CVCs had higher pain scores than those without (VAS: 6.8 ± 1.7 vs. 6.0 ± 1.8; p = 0.01). In contrast, they had significantly greater functional impairment (Lequesne index: 12.4 ± 4.2 vs. 10.3 ± 4.6; p = 0.004). Systemic NSAID use was significantly lower in patients with CVCs (31/98 (31.6%) vs. 33/60 (55.0%); p = 0.01), whereas no significant differences were observed for intra-articular corticosteroid injections or viscosupplementation.

Conclusion: Cardiovascular comorbidities are highly prevalent in patients with KOA and are associated with greater functional disability and reduced use of systemic NSAIDs. These findings emphasize the need for routine cardiovascular risk assessment and individualized therapeutic strategies to optimize the management of patients with knee osteoarthritis.

## Introduction

Knee osteoarthritis (KOA) is the most common form of osteoarthritis (OA) and a leading cause of pain and disability worldwide [1]. It predominantly affects women, with prevalence increasing with age, obesity, and sedentary lifestyle [2]. Clinically, KOA is characterized by joint pain, stiffness, functional limitation, and reduced mobility, which impair patients' quality of life [3].

In recent years, KOA has been increasingly recognized not only as a localized degenerative joint disease but also as a condition associated with systemic comorbidities, particularly cardiovascular comorbidities (CVCs) such as hypertension, diabetes, dyslipidemia, ischemic heart disease, and stroke [4]. These associations are explained by common risk factors such as obesity, aging, and physical inactivity, but also by shared biological mechanisms including low-grade systemic inflammation and metabolic dysfunction [5,6].

The coexistence of KOA and CVCs has major clinical implications. Patients with KOA and comorbid cardiovascular disease are at higher risk of severe pain, disability, and poorer quality of life [7]. Moreover, the presence of CVCs influences therapeutic decisions. For example, the use of nonsteroidal anti-inflammatory drugs (NSAIDs), often prescribed in KOA, is limited in patients with cardiovascular risk [8-9].

Despite this evidence, data on the prevalence and clinical associations of CVCs in Moroccan KOA populations are scarce. The primary objective of our study was to assess the prevalence of cardiovascular comorbidities among patients with KOA. The secondary objectives were to analyze the association between these comorbidities and pain severity, functional status, radiographic severity, and therapeutic management.

## Materials and methods

Study design and setting

We conducted a cross-sectional, observational study in the Rheumatology Department A of El Ayachi Hospital, University Rabat-Salé, Morocco. The study was carried out between July 10 and October 20, 2024. Reporting followed the Strengthening the Reporting of Observational Studies in Epidemiology (STROBE) guidelines for cross-sectional studies [9].

Participants

Eligible participants were adults (≥18 years) with primary KOA, diagnosed according to the Kellgren-Lawrence radiographic classification, which grades tibiofemoral OA severity from grade 0 (no radiographic features) to grade IV (severe, with large osteophytes, marked joint-space narrowing, and bone deformity) [10]. Exclusion criteria were secondary OA (post-traumatic or metabolic), inflammatory rheumatic diseases, rheumatoid arthritis (RA), spondyloarthritis, severe psychiatric or cognitive impairment, and incomplete clinical records.

Data collection procedures

Data were collected using a standardized case report form. Sociodemographic variables included age, sex, marital status, educational level, occupation, and type of medical coverage. Clinical characteristics of KOA included age at symptom onset, disease duration (calculated from symptom onset and from diagnosis), number of flares per year, walking distance (in meters), and radiographic severity according to the Kellgren-Lawrence classification.

Pain intensity was assessed using a 10-cm visual analog scale (VAS). Functional disability was evaluated using the Lequesne Algofunctional Index (LAI), a validated disease-specific questionnaire widely used to assess the severity of KOA [11]. The index evaluates pain or discomfort, maximum walking distance, and activities of daily living, with total scores ranging from 0 to 24; higher scores indicate greater functional impairment and disease severity. For the multivariable logistic regression analysis, a Lequesne Index >10 was used to define severe functional impairment, as this threshold corresponds to severe OA-related disability and has recognized clinical relevance in the interpretation of the Lequesne score.

Cardiovascular comorbidities (CVCs) were systematically assessed through medical history, physical examination, review of medical records, and laboratory investigations when available. Hypertension was defined as a blood pressure ≥140/90 mmHg on two separate measurements or current antihypertensive treatment. Diabetes mellitus was defined by a fasting plasma glucose level ≥126 mg/dL, HbA1c ≥6.5%, or current antidiabetic therapy. Dyslipidemia was defined as total cholesterol ≥200 mg/dL, LDL cholesterol ≥130 mg/dL, HDL cholesterol <40 mg/dL in men or <50 mg/dL in women, or current lipid-lowering therapy. Ischemic heart disease was defined as documented angina, previous myocardial infarction, or prior coronary revascularization. Heart failure was diagnosed according to established cardiology criteria. Cerebrovascular disease included a history of ischemic or hemorrhagic stroke, while peripheral arterial disease was defined by a history of intermittent claudication or previous peripheral arterial revascularization. Other comorbidities, including fibromyalgia, depression, menopausal status, and smoking status, were also recorded.

Therapeutic management during the six months preceding study inclusion was documented by reviewing medical prescriptions and patient interviews. Information was collected on the use of systemic analgesics, nonsteroidal anti-inflammatory drugs (NSAIDs; oral or topical), intra-articular corticosteroid injections, viscosupplementation, and rehabilitation or physiotherapy.

Laboratory data were recorded when blood tests performed within the previous three months were available and included fasting plasma glucose, HbA1c, total cholesterol, LDL cholesterol, HDL cholesterol, and triglyceride levels.

Outcomes

The primary outcome of the study was to determine the prevalence of CVCs among patients with KOA, thereby contributing to a better understanding of the systemic burden of OA, a disease increasingly recognized as involving low-grade systemic inflammation in addition to its degenerative features. Secondary outcomes included evaluating the association between CVCs and pain severity, as measured by a VAS, functional impairment assessed using the Lequesne Algofunctional Index, and radiographic severity according to the Kellgren-Lawrence classification. In addition, the study investigated the impact of cardiovascular comorbidities on therapeutic management, including the use of NSAIDs, intra-articular therapies (corticosteroid injections and viscosupplementation), and rehabilitation or physiotherapy.

Statistical analysis

Data were analyzed using Jamovi version 2.3. Continuous variables were expressed as mean ± standard deviation (SD) or median (interquartile range, IQR) as appropriate, and categorical variables as percentages. Normality was assessed using the Shapiro-Wilk test. Continuous variables were compared between groups (CVC+ vs. CVC−) using Student's t-test (for normally distributed data) or Mann-Whitney U test (non-parametric). Categorical variables were compared using the chi-square (χ²) test or Fisher's exact test when appropriate. Effect sizes were calculated to quantify the strength of associations. A multivariate logistic regression model was performed to identify independent predictors of functional impairment (Lequesne > 10), adjusting for age, sex, BMI, disease duration, and presence of CVCs. A p-value <0.05 was considered statistically significant.

Ethics approval

The study protocol was approved by the Ethics Committee for Biomedical Research at Mohammed V University, Faculty of Medicine and Pharmacy of Rabat (Approval number: 186/24). All participants provided written informed consent.

## Results

Patient characteristics

A total of 158 patients with KOA were included. The mean age was 62.3 ± 10.5 years, and 125 (79.1%) were female patients. The majority of the patients were overweight, with an average BMI of 29.4 ± 4.8. The median disease duration was 7 years (IQR 4-12). The mean VAS pain score was 6.4 ± 1.8, and the mean Lequesne Index was 11.2 ± 4.5 (Table 1).

**Table 1:** Baseline characteristics of patients with KOA BMI: body mass index; SD: standard deviation; IQR: interquartile range; KOA: knee osteoarthritis

Characteristics	Total (n = 158)
Age, years, mean ± SD	62.3 ± 10.5
Female sex, n (%)	125 (79.1)
BMI, kg/m², mean ± SD	29.4 ± 4.8
Disease duration, years, median (IQR)	7 (4-12)
Kellgren-Lawrence grade ≥ III, n (%)	102 (64.6)
VAS pain, mean ± SD	6.4 ± 1.8
Lequesne Index, mean ± SD	11.2 ± 4.5
Hypertension, n (%)	85 (53.8)
Diabetes mellitus, n (%)	46 (29.1)
Dyslipidemia, n (%)	41 (25.9)
Ischemic heart disease, n (%)	17 (10.8)
Stroke, n (%)	8 (5.1)
Peripheral arterial disease, n (%)	6 (3.8)

Prevalence of CVCs

Overall, 98 (62.0%) patients presented with at least one cardiovascular comorbidity. The most prevalent condition was hypertension, observed in 85 (53.8%) patients, followed by diabetes mellitus in 46 (29.1%) and dyslipidemia in 41 (25.9%). Ischemic heart disease was reported in 17 (10.8%) patients, while cerebrovascular disease (stroke) and peripheral arterial disease were less frequent, occurring in 8 (5.1%) and 6 (3.8%) patients, respectively (Table 1).

KOA patient characteristics by CVC status 

When comparing patients with and without CVCs, significant clinical differences were observed across several parameters. Patients with CVCs (n = 98) were markedly older (mean age 66.2 ± 9.1 years) than those without CVCs (n = 60) (58.1 ± 9.8 years; t(156) = 5.27, p < 0.001). Although the proportion of women was similar between groups (79/98 (80.6%) vs. 46/60 (76.7%); χ²(1) = 0.35, p = 0.56), individuals with CVCs tended to have a higher body mass index (30.1 ± 4.9 vs. 28.8 ± 4.7 kg/m²; t(156) = 1.64, p = 0.08). Disease duration was significantly longer in the CVC group (median 9 years (IQR 5-15)) compared to those without CVCs (6 years (IQR 3-10); Mann-Whitney U = 2221, p = 0.01). These baseline differences should be considered when interpreting the associations observed between CVCs and KOA severity. Radiographically, advanced KOA (Kellgren-Lawrence grade ≥ III) was more prevalent among CVC-positive patients (71/98 (72.4%) vs. 31/60 (51.7%); χ²(1) = 7.00, p = 0.006). Similarly, symptom severity differed between the groups: mean pain intensity on the VAS scale was 6.8 ± 1.7 in the CVC group compared with 6.0 ± 1.8 in those without comorbidities (t(156) = 2.81, p = 0.01). Although this difference was statistically significant, its clinical relevance should be interpreted with caution given its modest magnitude. Functional impairment was also significantly higher in the CVC group, as reflected by elevated Lequesne Index (12.4 ± 4.2 vs. 10.3 ± 4.6; t(156) = 2.94, p = 0.004) (Table 2).

**Table 2:** Differences between KOA patients with and without cardiovascular comorbidities CVC: cardiovascular comorbidities; SD: standard deviation; KOA: knee osteoarthritis; IQR: interquartile range; VAS: visual analog scale

Characteristics	Total (n=158)	With CVCs (n=98)	Without CVCs (n=60)	Test statistic	p-value
Age, years, mean ± SD	62.3 ± 10.5	66.2 ± 9.1	58.1 ± 9.8	t(156) = 5.27	<0.001
Female sex, n (%)	125 (79.1)	79 (80.6)	46 (76.7)	χ²(1) = 0.35	0.56
BMI, kg/m², mean ± SD	29.4 ± 4.8	30.1 ± 4.9	28.8 ± 4.7	t(156) = 1.64	0.08
Disease duration, years, median (IQR)	7 (4-12)	9 (5-15)	6 (3-10)	Mann-Whitney U = 2221	0.01
Kellgren-Lawrence grade ≥ III, n (%)	102 (64.6)	71 (72.4)	31 (51.7)	χ²(1) = 7.00	0.006
VAS pain, mean ± SD	6.4 ± 1.8	6.8 ± 1.7	6.0 ± 1.8	t(156) = 2.81	0.01
Lequesne Index, mean ± SD	11.2 ± 4.5	12.4 ± 4.2	10.3 ± 4.6	t(156) = 2.94	0.004

Association with therapeutic management

The use of systemic NSAIDs was significantly lower in patients with CVCs (31/98 (31.6%) vs. 33/60 (55.0%); χ²(1) = 8.44, p = 0.01). There was no difference in the prescription of intra-articular corticosteroid injections (28/98 (28.6%) vs. 24/60 (40.0%); χ²(1) = 2.20, p = 0.213) and viscosupplementation (22/98 (22.4%) vs. 21/60 (35.0%); χ²(1) = 2.97, p = 0.131) between the two groups, and the same was observed for rehabilitation (89/98 (90.8%) vs. 52/60 (86.7%); χ²(1) = 0.67, p = 0.485) (Table 3).

**Table 3:** Therapeutic management according to CVC status CVC: cardiovascular comorbidities

Therapeutic management	Total (n=158)	With CVC+ (n=98)	Without CVC (n=60)	Test statistic	p-value
NSAID, n (%)	64 (40.5)	31 (31.6)	33 (55.0)	χ²(1) = 8.44	0.01
Corticosteroid injections, n (%)	52 (32.9)	28 (28.6)	24 (40.0)	χ²(1) = 2.20	0.213
Viscosupplementation, n (%)	43 (27.2)	22 (22.4)	21 (35.0)	χ²(1) = 2.97	0.131
Rehabilitation, n (%)	141 (89.2)	89 (90.8)	52 (86.7)	χ²(1) = 0.67	0.485

Factors predicting functional impairment in KOA patients 

After adjusting for age, sex, BMI, and disease duration, the presence of CVCs was independently associated with severe functional impairment (Lequesne >10) (OR 1.9, 95% CI 1.1-3.4, p = 0.02) (Table 4).

**Table 4:** Multivariate logistic regression shows the predictors of severe functional impairment (Lequesne >10) BMI: body mass index; VAS: visual analog scale

Variable	OR	95% CI	p-value
Age >60 years	1.77	0.49-6.28	0.376
Female sex	0.86	0.06-10.77	0.905
Disease duration	1.01	0.89-1.12	0.919
BMI	1.09	0.94-1.26	0.250
Physical activity	0.94	0.40-2.20	0.894
VAS pain	1.18	0.92-1.52	0.190
Kellgren-Lawrence grade ≥ III	1.64	0.79-3.40	0.180
Prosthesis	3.14	0.13-73.88	0.478
Cardiovascular comorbidities (present vs absent)	1.90	1.10-3.40	0.020

It should be noted that some variables in the multivariate model, notably the presence of a joint prosthesis, showed markedly wide 95% confidence intervals (e.g., OR 3.14, 95% CI 0.13-73.88). This imprecision reflects the small number of patients within these specific subgroups rather than a true absence of association, and estimates for these variables should therefore be interpreted with caution. 

## Discussion

In this cross-sectional study of 158 Moroccan patients with KOA, we found a high prevalence of CVCs, reaching 62.0% (98/158), with hypertension, diabetes, and dyslipidemia being the most frequent. Patients with CVCs reported higher pain scores, greater functional limitation, and more advanced radiographic severity compared to those without comorbidities. The higher prevalence of advanced Kellgren-Lawrence grades among patients with CVCs further supports the association between structural joint damage and cardiometabolic burden, although longitudinal studies are needed to determine the temporal relationship between these findings. These findings provide new regional data on the intersection between KOA and cardiovascular diseases, an increasingly recognized but underexplored relationship in low- and middle-income countries [12].

Our results are consistent with global trends. OA is among the leading causes of disability worldwide, with an increasing burden attributed to population aging and obesity [1,13]. A Brazilian cross-sectional study reported a 50% prevalence of hypertension and a 20% prevalence of diabetes among OA patients [14], comparable to our findings. Likewise, a population-based German cohort showed that comorbidities, especially cardiometabolic ones, significantly worsen pain and functional limitations in OA [15].

Globally, several population-based cohorts have reported the same association between OA and cardiovascular disease. A study found that metabolic comorbidities such as obesity, hypertension, and diabetes were independent risk factors for the onset of OA [2]. Another study confirmed that OA patients had a significantly higher incidence of cardiovascular events compared to matched controls, even after adjusting for traditional risk factors [4,7]. Moreover, a study demonstrated in a meta-analysis that OA was associated with increased all-cause and cardiovascular mortality, further emphasizing the systemic impact of the disease [5].

Although our cross-sectional design does not allow causal inference, several biological mechanisms have been proposed to explain the observed association between KOA and CVCs. The relationship is likely complex and multifactorial. Both conditions share common risk factors--aging, obesity, and sedentary lifestyle--but accumulating evidence points to deeper biological connections [3,6]. Berenbaum proposed the concept of “inflammatory osteoarthritis,” emphasizing the role of systemic low-grade inflammation in OA pathogenesis [6]. Adipokines, interleukin-6, and C-reactive protein, key mediators of inflammation, contribute not only to cartilage degradation but also to endothelial dysfunction and atherogenesis [16].

A study further detailed the “metabolic-inflammatory” link between OA and cardiovascular disease, describing how chronic inflammation within adipose tissue promotes both joint damage and vascular injury [16-17]. These findings support the hypothesis that, in some patients, OA may be associated with systemic low-grade inflammation and metabolic dysregulation rather than representing only a local mechanical disorder.

In our study, the Lequesne index was used to quantify the functional impact of KOA on patients' daily lives. This validated instrument allowed us to capture not only the degree of pain and stiffness but also the extent of mobility restriction experienced by our population. The mean scores observed in our cohort reflect a moderate to severe level of functional disability, consistent with the chronic nature of the disease and the long median duration of symptoms. Interestingly, patients presenting with CVCs exhibited significantly higher Lequesne scores, suggesting an association between poorer joint function and the presence of CVCs. However, this association may also be influenced by differences in age, disease duration, and body mass index between the two groups. This association underscores the interconnection between functional decline, inflammation, and cardiovascular risk in patients with KOA.

Functional impairment caused by knee pain may also contribute indirectly to cardiovascular risk. In patients with KOA, the restriction of daily mobility may promote weight gain, central obesity, and insulin resistance, potentially contributing to endothelial dysfunction. Moreover, chronic pain activates the sympathetic nervous system, increasing blood pressure and vascular stiffness [4]. Adipokines such as leptin, resistin, and visfatin, secreted by hypertrophic adipose tissue, exert catabolic and pro-inflammatory effects on cartilage, subchondral bone, and synovium. These mediators also induce endothelial dysfunction and oxidative stress within the vascular system [18]. These mechanisms may contribute to a bidirectional interaction between mechanical limitation and metabolic disorders, fostering a chronic low-grade inflammatory state that perpetuates both joint degradation and cardiometabolic disease, highlighting the biological continuum between articular and cardiovascular inflammation. Furthermore, the metabolic burden of obesity and insulin resistance amplifies mitochondrial dysfunction and systemic oxidative stress. This environment promotes sarcopenia, alters muscle strength, and reduces proprioceptive capacity, thereby worsening functional disability and frailty in older adults with KOA [19]. Taken together, these observations suggest that OA and CVCs may share common inflammatory and metabolic pathways. However, the cross-sectional design of our study does not allow conclusions regarding the direction or causality of these relationships. Recent work further supports this integrative concept, linking OA progression to metabolic syndrome, hypertension, and atherosclerotic disease [19,20].

From a therapeutic perspective, our study demonstrates that the coexistence of CVCs substantially influences the management strategy of KOA. The use of NSAIDs was significantly reduced among patients with cardiovascular conditions, reflecting well-founded concerns about their potential impact on blood pressure, renal function, and thrombotic risk. This observation aligns with both the 2019 Osteoarthritis Research Society International (OARSI) guidelines and the 2018 European Society of Cardiology/European Society of Hypertension (ESC/ESH) hypertension guidelines, which recommend caution with systemic NSAIDs in patients at cardiovascular risk [17,21].

Topical NSAIDs, intra-articular corticosteroid injections, and non-pharmacological modalities are preferred for pain control in these patients. Adjunctive therapies, such as acetaminophen, low-dose tramadol, or duloxetine, may offer symptomatic benefit with a safer cardiovascular profile. Additionally, structured physiotherapy programs focused on quadriceps strengthening, balance training, and aerobic conditioning are critical to break the vicious cycle of inactivity and metabolic decline. Exercise therapy, when properly supervised, improves both joint function and cardiovascular fitness without exacerbating pain or inflammation [22].

The complexity of this dual burden necessitates a multidisciplinary approach that integrates rheumatologic, cardiologic, and metabolic follow-up. Coordination between specialists ensures optimized pharmacologic safety, individualized physical rehabilitation, and management of modifiable cardiovascular risk factors such as obesity, hypertension, dyslipidemia, and diabetes. This integrated care model not only improves symptom control but also reduces hospitalization rates and long-term morbidity.

Strengths

One of the main strengths of our study is that it was conducted in Morocco, a developing country, which provides a unique context compared to studies performed in Europe or North America. The dietary habits, lifestyle factors, and genetic backgrounds of our population differ substantially from those in Western countries, allowing for the examination of disease characteristics and risk factors in a distinct setting.

To our knowledge, this is one of the few studies to address this topic in North Africa. It adds valuable data to a region where epidemiological research is scarce. In addition, our study included a relatively large sample for a single-center observational study, with 158 patients, compared with 91 patients in a previous cross-sectional study assessing comorbidities in OA [14]. We also used validated clinical and radiographic assessment tools, comprehensive data collection, and multivariate analysis to strengthen the reliability of our findings.

Limitations

However, some limitations should be acknowledged. The single-center design may limit the generalizability of our findings, and the cross-sectional nature precludes causal inference. It remains uncertain whether CVCs exacerbate OA severity or whether OA contributes to increased cardiovascular risk through inflammation and reduced mobility. Longitudinal studies are needed to explore the temporal relationship between these conditions. In addition, some variables included in the multivariable logistic regression analysis were based on relatively small subgroup sizes, which may have contributed to statistical instability and wide confidence intervals. Therefore, these findings should be interpreted with appropriate caution. Additionally, we did not assess adipokines or other inflammatory biomarkers, such as IL-6 and adiponectin, nor did we perform advanced imaging techniques. These measures could have provided further insight into the inflammatory and metabolic pathways linking KOA and CVCs, particularly the shared mechanisms involved in chronic low-grade inflammation and atherosclerosis.

Implications for future research

Future research should focus on longitudinal designs assessing the progression of both OA and cardiovascular disease, as well as interventional studies evaluating whether optimal cardiovascular risk management improves pain, function, or radiographic outcomes. Investigations into the role of metabolic and inflammatory biomarkers could also provide mechanistic insights. Moreover, in the Moroccan context, multicenter collaborations would strengthen the external validity of these observations and help shape region-specific guidelines.

## Conclusions

CVCs are highly prevalent among Moroccan patients with KOA and are associated with greater pain, functional disability, and radiographic severity. They also influence therapeutic strategies, reducing NSAID use and increasing intra-articular and rehabilitation approaches.

These findings underline the need for an integrated management strategy addressing both joint disease and cardiovascular health in KOA patients.
